# Photoinduced Charge Transfer and Vibronic Coherence in CdSe Quantum Dots with Methyl Viologen Acceptors

**DOI:** 10.1021/acs.jpcc.6c02147

**Published:** 2026-04-22

**Authors:** Nila Mohan T. M., Shanu A. Shameem, Chase H. Leslie, Caitlin V. Hetherington, Benjamin G. Levine, Warren F. Beck

**Affiliations:** † Department of Chemistry, 3078 Michigan State University, 578 S. Shaw Lane, East Lansing, Michigan 48824 United States; ‡ Institute for Advanced Computational Science and Department of Chemistry, 12301 Stony Brook University, Stony Brook, New York 11794, United States

## Abstract

We show herein that photoinduced charge transfer from CdSe quantum dots (QDs) to surface-bound methyl viologen (MV^2+^) acceptors is mediated by a vibronically coherent, nonadiabatic mechanism. Broadband multidimensional electronic spectra and an analysis of coherences show that a mixed QD–MV charge-transfer (CT) state is populated on the <50 fs time scale after optical preparation of the X3 (1P_3/2_–1P_e_) state, well prior to the appearance of the one-electron photoreduced ground state (MV^+•^). A partial redistribution of charge from the core of the QD to the acceptor is revealed by excited-state coherences of an out-of-plane vibrational mode local to MV^2+^ and of a low-frequency mode mixing a MV^2+^ mode with the longitudinal optical (LO) phonon of the QD core. The ultrafast damping of these coherences indicates that excited-state wavepackets travel from the optically prepared, Franck–Condon structure through a conical intersection to reach the CT state. These results suggest that vibronically coherent processes generating CT intermediates can be exploited to improve the efficiency of QD-based solar cells and photocatalysts.

## Graphical abstract

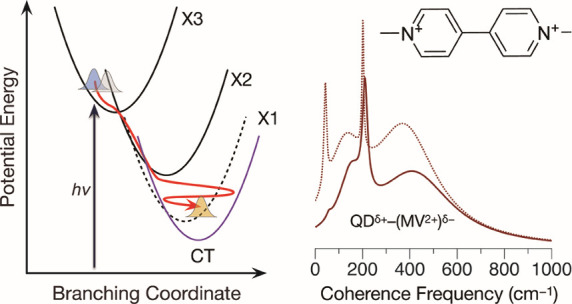

## Introduction

The efficiency of solar cells^1,2^ and photocatalysts^3^ employing semiconductor quantum dots (QDs) as light-harvesting structures is lower than theoretically possible^4,5^ because a substantial fraction of the excitation energy captured from solar photons is rapidly dissipated by hot-carrier cooling, the intraband nonradiative relaxation from the optically prepared exciton state to the band-edge state.^6–11^ There is considerable evidence that the vibrations of the surface-capping ligands of colloidal QDs^12^ play an active role in the dynamics of hot-carrier cooling by capturing excitation energy on the sub-ps time scale.^13–18^

In a recent series of studies using broadband multidimensional electronic spectroscopy (2DES and 3DES), we showed that the excitation energy of the X3 (1P_3/2_–1P_e_) state in hexadecylamine (HDA)- and oleate-capped CdSe QDs is converted to ligand vibrations on the <50 fs time scale.^19–21^ Global models of the broadband 2DES spectrum observed with ∼7 fs excitation pulses indicate that the X3 state relaxes nonradiatively following optical excitation to the X2 (2S_3/2_–1S_e_) and X1 (1S_3/2_–1S_e_) states on a similar time scale as the damping of excited-state vibronic coherences. This phenomenon reveals that the conversion of excitation energy to ligand vibrations involves a vibronically coherent passage of excited-state wavepackets through a cascade of conical intersections (CIs) between the exciton states of the QD. The role played by CIs in the photochemistry of organic molecules^22–27^ is well-known, but it is increasingly evident that CIs can be involved in ultrafast nonradiative decay processes in materials.^28^ The existence of CIs in QDs and the role of ligand-derived vibrational modes in hot-carrier cooling have been recently probed in calculations of alkylamine-^29^ and alkylcarboxylate-capped^30^ Cd_33_Se_33_ nanoclusters. Vibrational modes local to the organic ligands apparently serve as the *branching* (or tuning and coupling) modes,^31^ which control the energy gap and the strength of interaction of two diabatic potential energy surfaces.^24,32,33^ This is a *molecular* picture for the photochemistry of QDs, in which the ligand vibrations are active participants in the photophysics, not just channels for the incoherent dissipation of excitation energy.

These experimental and theoretical findings raise an important question about the mechanism of photoinduced charge transfer (CT) and of triplet–triplet excitation energy transfer (TTET) to surface-bound acceptors in QD systems. In a conventional picture for the photophysics, the X1 band-edge state resulting from hot-carrier cooling would be expected to thermalize prior to electron transfer on the ps time scale, dissipating the excess excitation energy above the band edge. In the oleate- and HDA-capped CdSe QDs, intramolecular vibrational redistribution (IVR) processes on the ∼200 fs time scale follow the initial, ultrafast electronic process mediated by coherences of the surface-bound organic ligands. In the presence of electron or excitation energy acceptors on the surface of a QD, however, it is possible that an intermediate with a partial redistribution of charge from the QD core to the acceptor would be favored, initiating CT and quenching the initial excitation. In this contribution, we have tested this hypothesis in a study of photoinduced CT in CdSe QDs to surface-bound methyl viologen dication (MV^2+^) acceptors. Using broadband multidimensional electronic spectroscopy and an analysis of coherences, we have detected the vibronically coherent formation of a CT state with a mixed character, with oscillator strength from the QD and vibrations of the core of the QD detected alongside the local vibrations of MV. This finding has important implications for the design of QD-based materials for use in solar cells and in photocatalysis because the CT state is formed on a very short time scale compared to thermalization in the band-edge state.

## Experimental Section

CdSe QDs with oleate ligands were prepared and characterized by the Van Patten and Zhang laboratories at Middle Tennessee State University. The QD preparations used in the present study were discussed previously.^20,21^ Methyl viologen dichloride hydrate (MV^2+^, Sigma–Aldrich 856177) was used as received. To prepare the MV-treated samples used here in studies of photoinduced CT, a methanol solution of MV^2+^ was added to the oleate-capped QDs in chloroform to establish a 1:50 QD:MV ratio, in line with a previously reported procedure.^34^

Absorption spectra of the oleate-capped and MV-treated CdSe QDs were measured in a Shimadzu UV-2600 spectrometer at ambient temperature (293 K). Photoluminescence spectra were recorded with a home-built fluorescence spectrometer^35^ in which the excitation light is provided by a broadband LED (Thorlabs M530L4) filtered by a double monochromator (Spectral Products AG1200–00500–303) in an additive configuration, providing a spectral bandpass of 2 nm. Emission was detected with a 4 nm spectral bandpass and magic-angle polarization by an Acton Research SP-150 spectrograph and an Oxford Instruments Andor Newton 940-BV CCD detector. An Ocean Optics HG-1 lamp was used to calibrate the excitation monochromator and emission spectrograph; an Ocean Optics HL-3 plus-CAL reference lamp was used to calibrate the absolute spectral response of the detection system. The apparatus was controlled by LabVIEW (National Instruments) routines. The concentration of the QDs was adjusted with addition of chloroform solvent to obtain an absorbance of 0.3 measured at the wavelength of the band-edge transition, 565 nm, for an optical path length of 1 mm in a quartz cuvette. The QD samples were held in a fused silica cuvette with a 1 mm optical path length.

Broadband two- and three-dimensional electronic spectra (2DES and 3DES) were acquired at ambient temperature (293 K) using the instrumentation and methods discussed previously,^36,37^ with a two-beam, pump–probe spectrometer employing adaptive pulse shaping^38^ and phase-sensitive detection.^39^ Broadband (∼520 to 700 nm) laser excitation pulses were obtained from a noncollinear optical parametric amplifier (NOPA), which was pumped by an amplified Yb laser. The duration of the pump and probe pulses at the sample’s position was determined separately by the pulse shaper in each beam using MIIPS scans^40^ to be 7.0 fs. The pulse energy for each of the three laser pulses in the stimulated photon echo sequence was adjusted using neutral density filters to 3.75 nJ, with a 100-kHz repetition rate, which results in an average incident power of 1.125 mW. The focused pump and probe spots on the sample were ∼100 μm in diameter, as measured by a beam profiling camera. Figures S1–S3 in the Supporting Information provide residual phase plots, a SHG-FROG^41^ spectrogram, and an interferometric autocorrelation signal, which were measured with the pulse shapers.^40^ Data sets of the control, oleate-capped QDs were reported previously in a manuscript presenting an analysis of coherences.^20^ The 2DES spectra reported there and in the present manuscript are the sum of those from the rephasing and nonrephasing nonlinear optical response pathways; phase cycling of the excitation pulses was not performed.^38^

Global and target models of the 2DES spectra were determined with the CarpetView program (Light Conversion). Confidence intervals for the time constants in the global models are estimated for the <1 ps components from separate models of the individual *T* delay scans in the data sets. The *T* axis was sampled at 2.5 fs steps over the −20 to 20 fs range, with 5 fs steps then used up to 400 fs and 10 fs steps up to 800 fs. 500 fs and 1 ps steps were used over the 1–2 ps and 2–10 ps ranges, respectively. Because the sampling of the *T* axis is relatively sparse at long delays, we only provide rough estimates for the time constants for the ps and slower components, including that for ground-state recovery. Linear prediction, singular value decomposition (LPSVD) models of coherences were obtained with a Julia program we ported from the MATLAB code supplied by Champion and co-workers.^42,43^ LPSVD power spectra were calculated by scaling the fitted amplitudes with normalized (unit-area) Lorentzian line shapes, which allows one to compare the spectra with conventional Fourier transform power spectra.

## Results and Discussion

For the studies reported in the following, we used samples of CdSe QDs synthesized with oleate surface-capping ligands from the same preparation used previously in studies of vibronic coherences and hot-carrier cooling.^20^ For studies of photoinduced CT, MV^2+^ was added to the oleate-capped QDs to quench 70% of the photoluminescence (PL) yield observed at ambient temperature (293 K). Broadband 2DES spectra of the MV^2+^-treated QDs were recorded with 7 fs laser pulses having intensity spectra spanning the absorption transitions to the lowest three exciton states, X1–X3.

At very short probe delays *T*, the 2DES spectra from the MV^2+^-treated QDs shown in Figure 1 exhibit a set of partially resolved peaks along the diagonal, corresponding to ground-state bleaching (GSB) and stimulated emission (SE) signals at the energies of the X1–X3 states, and an additional peak appears below the energy of X1 due to the fine structure.^44,45^ The GSB and SE signals are superimposed on a broad excited-state absorption (ESA) band due to transitions from singly to doubly excited exciton states spanning the detection axis from the energy of the photoluminescence (PL) spectrum through to the X3 region. Owing to the low excitation energies used in the present experiments, these signals arise from much less than one electron–hole excitation per QD. Cross peaks due to SE pathways begin to develop below the diagonal of the spectrum over the <20 fs time scale, indicating that population is being transferred from the X3 state to lower energy states via an ultrafast process. At the same time, the spectrum broadens in the antidiagonal direction owing to homogeneous and inhomogeneous line broadening. The initial phase of population relaxation is complete in ∼200 fs, which results in SE character in a broad cross peak independent of the excitation energy across the spectrum. The cross peak spans the energies of the lowest two exciton states, X1 and X2, and of the photoluminescence (PL) spectrum.

**Figure 1:**
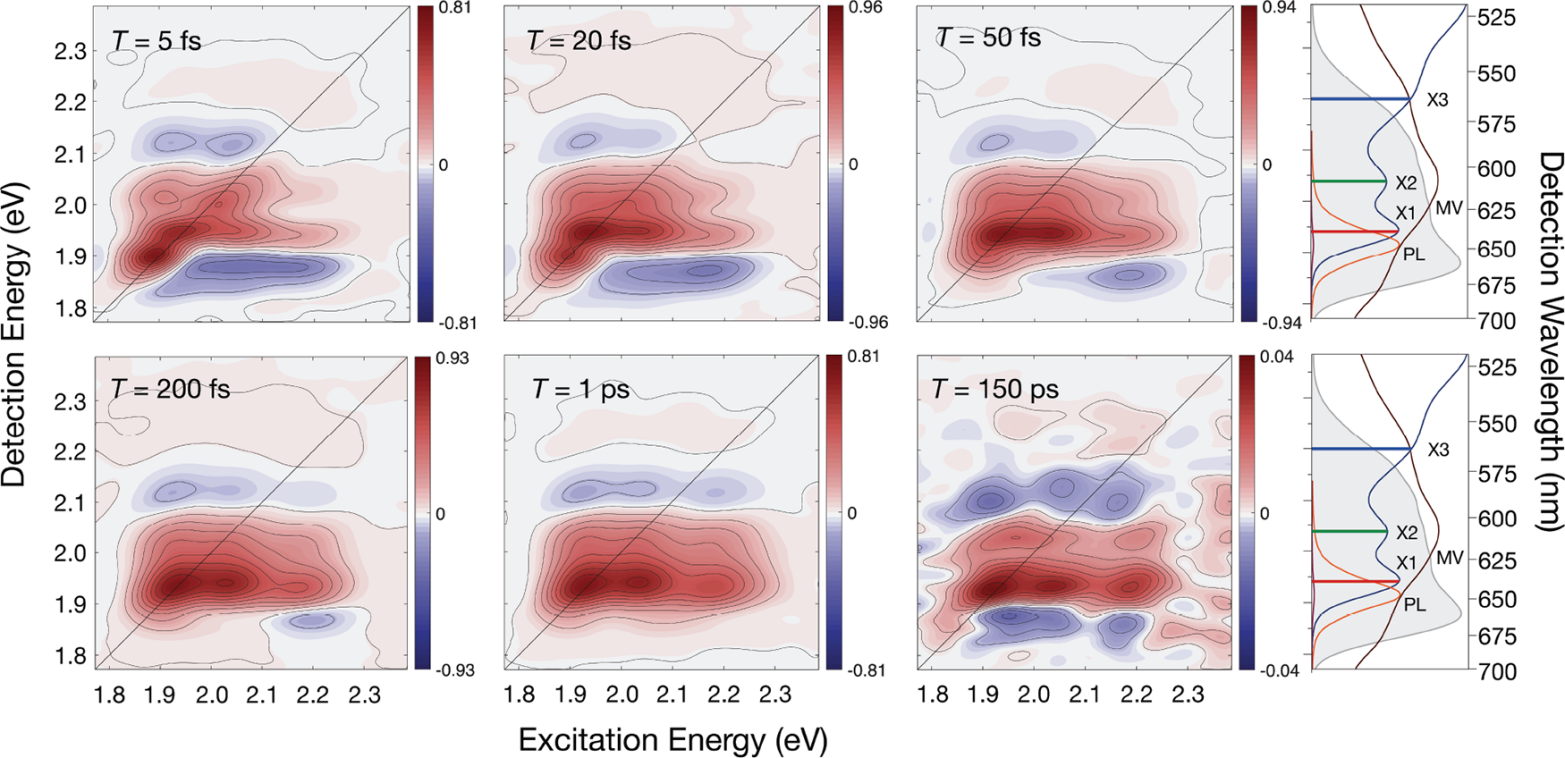
2DES spectra from CdSe QDs treated with methyl viologen dication (MV^2+^) at several probe delays *T*. The side panels on the right show the linear absorption oscillator strength spectrum (ε/ν, blue), the photoluminescence spectrum of the unquenched oleate-capped QDs (PL, orange), the residual photoluminescence spectrum of the MV-quenched, oleate-capped QDs (dark red), the ground-state absorption spectrum of MV^+•^ (dark purple), and the intensity spectrum of the ∼7 fs laser pulses (gray shaded). Blue, green, and red bars mark the energies of the X1–X3 exciton states, as estimated from the partially resolved peaks in the absorption spectrum. The 2DES spectra are shaded with respect to the adjacent color bars, which indicate the scaling relative to the maximum amplitude in the data set. The 2DES spectra have not been corrected for the spectra of the excitation and detection laser pulses.

The 2DES spectrum recorded at the probe delay of 150 ps shows that the ground-state one-electron photoproduct, MV^+•^, has been formed at the expense of the initial QD excitation due to the full transfer of an electron from the QD core to the MV acceptor. A weak cross peak remains, spanning the detection energies of the X2 and X1 states and the PL spectrum, which indicates that a fraction of the photoexcited ensemble of QDs was not quenched. However, a negative going, photoinduced absorption signal matching the absorption spectrum of ground state MV^+•^ accounts for the remaining yield of the initial excitation. Note that the molar extinction coefficient near the 606 nm absorption maximum of MV^+•^ is only 13,700 M^–1^ cm^–1^.^46^ The quantum yield for the photoinduced CT reaction is essentially unity,^47,48^ so the amplitude of the residual cross peak from the QD is in line with the fraction of unquenched QDs for the concentration of MV^2+^, as set up for this experiment.

The time evolution of the 2DES signal amplitudes due to population relaxation during the hot-carrier cooling and photoinduced CT processes in the presence of MV^2+^ can be obtained from a global and target model. Figure 2 reports a global model for a slice of the 2DES spectrum at the excitation energy for transitions to the X3 state. The signal amplitude, $A(E_{\det},T)=\underset{n}{\sum }P_{n}(T)S_{n}(E_{\det})$ , is fit here as a function of the delay time *T* with respect to the detection energy *E*_det_ by a linear combination of a set of *n* basis spectra, *S*_*n*_(*E*_det_), which are termed evolution-associated difference spectra (EADS).^49^ These spectra are scaled by the time-dependent populations in each of the spectrokinetic species, *P*_*n*_(*T*), in a proposed kinetic model. In the present case, a minimally determined, approximate model for the response of the MV^2+^-treated CdSe QDs out to a probe delay *T* of 100 ps requires six spectrokinetic species along a linear nonradiative decay pathway. The number of required species was determined by inspection of the residual signal amplitude (signal–model) in a series of *A*(*E*_det_, *T*) transients sampled near the diagonal and at the detection energies of the X1 and PL states.

**Figure 2:**
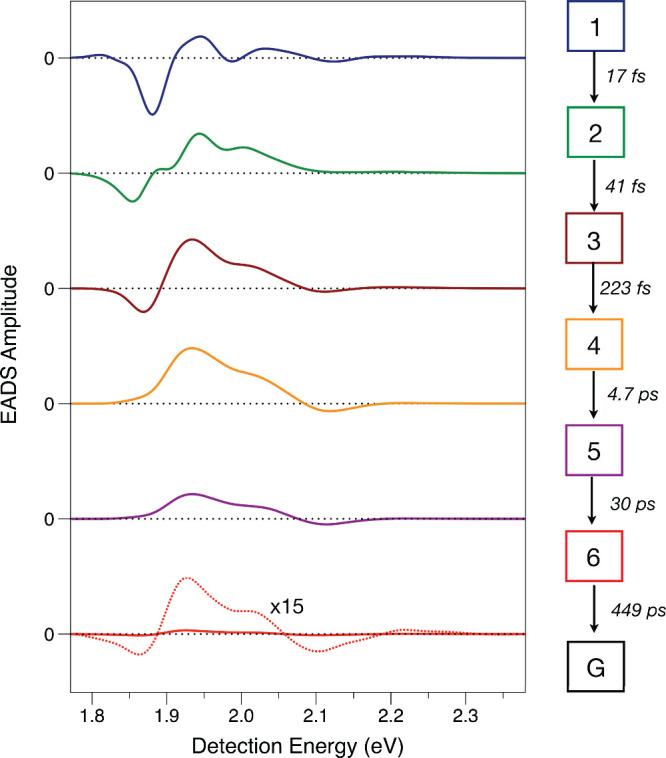
Evolution-associated difference spectra (EADS) from a global model for the slice of the 2DES spectrum at the excitation energy of the X3 transition of CdSe QDs treated with methyl viologen dication (MV^2+^). The spectra correspond to the six spectrokinetic species shown in the legend to the right of the spectra for a linear nonradiative decay pathway with the indicated time constants. Component 1 is for the instantaneously produced species prepared optically by the excitation pulses, and G indicates the original, equilibrium ground state. The EADS for the sixth spectrokinetic species is also shown as a dashed spectrum with ×15 scaling of the amplitude.

The EADS shown in Figure 2 for the MV-treated QDs resemble those reported recently^21^ for the untreated, oleate-capped QDs. The initial steps in the sequence of spectrokinetic species, with time constants of 17 ± 2 and 41 ± 4 fs, correspond to electronic relaxation of the X3 state, which is accompanied by red-shifting of the SE to lower energies spanning the X2 and X1 states. This process converts the excitation energy of the X3 state in the conduction band to vibrations, either of the surface-capping ligands or the MV acceptors, via a coherent nonadiabatic mechanism.^29,30^ This conclusion is supported by an analysis of coherences, which follows below. Subsequently, with a time constant of 223 ± 11 fs, the GSB/SE signal at X1 exhibits a modest increase in intensity as the ESA band appears to shift to the blue, with the negative signal amplitude at 1.875 eV disappearing as that at 2.125 eV increases. In the global model for the oleate-capped QDs, we assigned this step to a vibrational cooling process mediated by IVR, which would dissipate excess vibrational energy in the active modes, initially to low frequency ligand vibrations.^21^ The time constant observed here is characteristic of IVR in large organic molecules^36,50,51^ and in coordination complexes.^52–54^

The formation of MV^+•^ then follows on the ps time scale as a biexponential process. The global model includes two successive steps that decrease the overall intensity of the EADS spectrum with time constants of 4.7 and 30 ps, without incurring a significant change of shape or position with respect to the detection energy axis. A similar pair of time constants was observed previously in studies of photoinduced CT to MV^2+^ in CdSe QDs^55^ and in CdS QDs,^34^ although a 70 fs time constant was reported for smaller CdSe QDs.^56^ The faster of the two time constants observed in the CdS QDs, 3 ps, results in the formation of a broad, photoinduced absorption “shelf” attributed to transitions to midgap states,^34^ but a broader spectral line shape is not observed here in these CdSe QDs. The EADS spectrum of the sixth spectrokinetic species, the product of the 30 ps process, includes negative-going photoinduced absorption bands from the MV^+•^ product and a weak residual signal from the X1 state of the QD, the latter similar to the EADS of the fourth species. This signal arises from the fraction of unquenched QDs in the sample.

Although the 2DES spectra of the control oleate-capped and the MV^2+^-treated QDs appear to be very similar at short delays *T*, the transition state for the photoinduced CT reaction in the latter is evidently crossed during the initial electronic relaxation steps on the <50 fs time scale. This conclusion is based on the finding that the excited-state wavepacket motions accompanying the conversion of the excitation energy of the X3 state are more rapidly damped in the presence of MV^2+^. Additionally, an analysis of the accompanying vibronic coherences at low energies on the detection axis indicates that an intermediate CT state with mixed QD and MV character is promptly produced, rather than the band-edge X1 state observed in the control, oleate-capped QDs.

Figure 3 compares amplitude transients sampled at three coordinates of the 2DES spectra of the control, oleate-capped and MV^2+^-treated QDs at the excitation energy of the X3 state. As reported previously,^21^ a comparison of the responses of HDA- and oleate-capped QDs at the diagonal of the spectrum indicates that ultrafast electronic relaxation from the optically prepared X3 state strongly damps the initial modulated portion of the 2DES signal response. Inspection of the (X3,X3) transients near the diagonal indicates that the signal amplitude decreases perhaps a factor-of-two more rapidly in the MV^2+^-treated sample due to an ultrafast process. The MV^2+^-treated sample exhibits a decreased signal amplitude at the maximum following the instrument-response limited rise, a faster initial decay from the maximum, and a shorter *T* delay for the zero-amplitude crossing compared to the oleate control.

**Figure 3:**
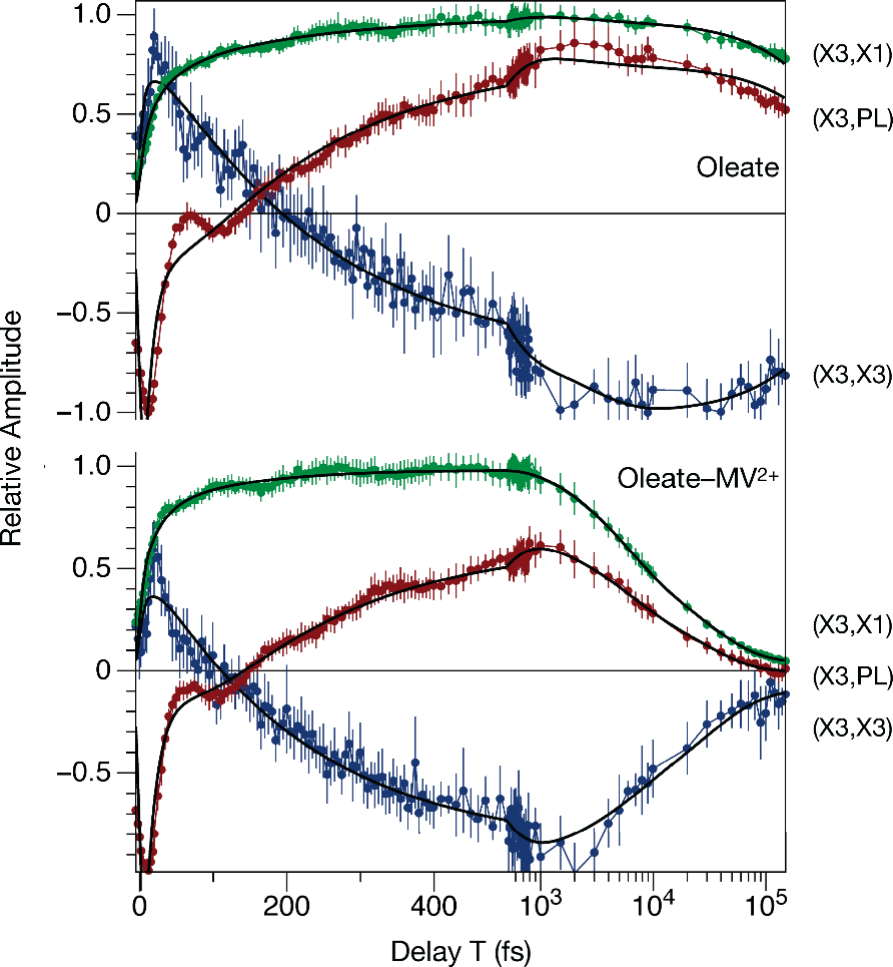
Amplitude transients sampled at the (X3,X3), (X3,X1), and (X3,PL) coordinates marked in Figure 1 of the 2DES spectra for the control, oleate-capped, and MV^2+^-treated CdSe QDs. Each transient is superimposed on smooth curves plotting the fitted amplitude from the global model shown in Figure 2; the thin vertical bars indicate 95% confidence intervals for each amplitude data point. The top panel for the control, oleate-capped QDs is adapted with permission from ref 20 Copyright 2026 AIP.

A comparison of the (X3,PL) transients for the oleate-capped and MV^2+^-treated QDs observed in the PL region of the 2DES spectra substantiates the conclusion that the presence of MV^2+^ accelerates and more rapidly damps the wavepacket motions that initiate photoinduced CT. Figure 4a presents a comparison of the modulated residual signals at (X3,PL) in the oleate-capped and MV^2+^-treated QDs after subtraction of the global model. The initial recurrence of the signal amplitude that contributes the intense “quantum beat” in the transient is perhaps 20 fs earlier on the delay *T* axis in the residual of the MV^2+^-treated sample than in the residual of the oleate control. This results in a phase-shifted response, with the subsequent modulation features observed earlier in the MV^2+^-treated sample. These observations indicate that wavepackets arrive in the product CT state more rapidly than the X1 state is produced in the control.

**Figure 4:**
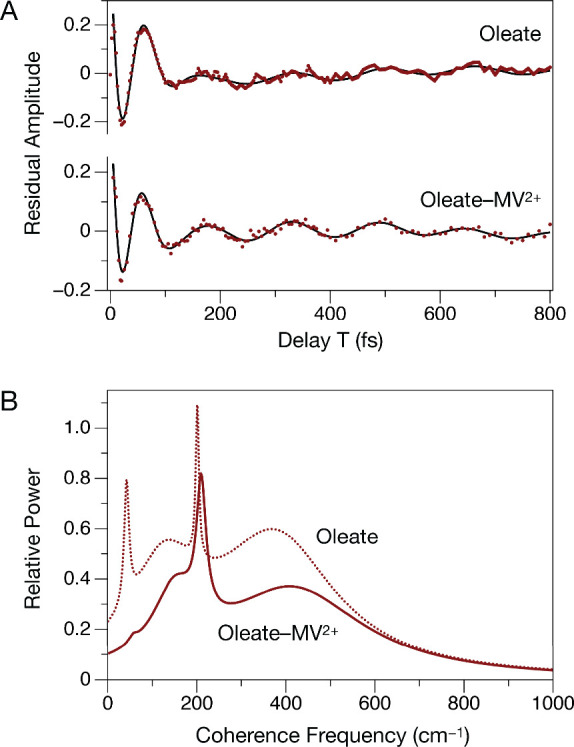
(A) Residual transients (data–global model) for the (X3,PL) transients shown in Figure 3 for the control oleate-capped and MV^2+^-treated CdSe QDs. The data points are superimposed with a model (black curve) incorporating four damped cosinusoids, which was obtained from a linear prediction, singular-value decomposition (LPSVD) analysis. Tables S1 and S2 list the model parameters for the four components. (B) Power spectra for the four modulation components, as plotted using the LPSVD model parameters.

Figure 4b models the residual transients of the MV^2+^-treated and oleate controls using linear-prediction, singular value decomposition (LPSVD) analysis^42,43,57–59^ to isolate the principal cosinusoidal modulation components and to estimate their damping time constants. An analysis of the dependence of the residual chi-square on the number of components, essentially as recommended previously, limits the models shown here to only four components.^59^ The models show that the rapidly damped excited-state coherences observed following the initial electronic relaxation process in the two samples carry a different vibrational mode content, and the mode assignments support the conclusion that an altered electronic structure due to partial CT from the QD’s core is produced on an ultrafast time scale but prior to the full reduction of MV^2+^.

The strongest modulation components carried by the (X3,PL) residual transient for the control, oleate-capped QDs exhibit frequencies of 126 and 375 cm^–1^. These components were assigned in the recent manuscript on the coherences in the oleate-capped QDs^20^ to a mixture of the LO phonon with a carboxylate mode and to a wagging vibration of the OCO moiety of the carboxylate and/or a bending vibration of the alkyl carboxylate CCO segment. The damping times determined by the LPSVD analysis are 58 and 32 fs, which indicate that the wavepacket motion with respect to these modes is being damped during the ultrafast electronic relaxation process noted above. The LPSVD power spectra shown in Figure 4b exhibit broad line shapes due to these components.

Additionally, the LPSVD model for the (X3,PL) transient for the oleate-capped QDs returns slowly damped components at 42 and 201 cm^–1^, with damping times of 810 and 1230 fs, respectively. These features contribute the two sharper peaks in the LPSVD power spectra. The long damping times here are consistent with an assignment to ground-state wavepacket motions launched by stimulated Raman pathways.^60^ The 201 cm^–1^ component is attributed to the LO phonon of the CdSe QD’s core. As discussed in detail previously,^20^ a progression of peaks is observed along the detection frequency axis of a Fourier transform oscillation map at this frequency, with the peak spacing indicating that the LO phonon is mixed with a oleate ligand-specific mode. Figure S4 compares the oscillation maps for two delay *T* windows, 5–100 and 100–500 fs, the latter emphasizing the more slowly damped components.

In contrast, the (X3,PL) residual transient for the MV^2+^-treated QDs exhibits rapidly damped coherences at somewhat higher frequencies, 150 and 415 cm^–1^, with damping times of 90 and 27 fs, respectively. While the character of the 150 cm^–1^ mode very likely involves a mixture with the core of the QD, given that the frequency is lower than that of the LO phonon, the 415 cm^–1^ component apparently corresponds to a local out-of-plane vibrational mode of the pyridinium rings of MV^2+^. This assignment is based on a comparison to the assigned normal coordinates of bands observed in this frequency region in the IR and resonance Raman spectra of MV^2+^ and of MV^+•^.^61^ An out-of plane vibration would modulate the permanent dipole moment of the acceptor with respect to the QD–MV^2+^ complex, given the expectation that MV^2+^ adsorbs to a QD with its rings flat on the surface.^47^ The natural frequency for this vibration would be expected to be somewhat lower than that for an unbound MV species, owing to a contribution to the reduced mass from the QD. In-plane deformations of the methyl group of MV^2+^ cannot be excluded at this point from consideration, owing to the broad line shape arising from the very rapid damping of the coherence, but this type of motion is assigned^61^ to still lower frequencies than observed here and would not be expected to contribute to the tendency to accept an electron from the QD. Note also that the Fourier transform oscillation maps for the MV^2+^-treated QDs shown in Figure S4 exhibit a number of additional modulation components in the PL region of the detection axis that are not observed in the corresponding maps from the oleate-capped control QDs. This observation suggests that additional vibrations of the MV^2+^ acceptor accompany the CT state. For example, a distinct peak at ∼1600 cm^–1^ is likely due to the C=C and C=N stretching vibrations of the pyridinium rings of MV^2+^, which would be displaced by addition of electron density to their delocalized π* orbitals.

These results lead to the picture we have for the dynamics in MV^2+^-treated QDs depicted in the cartoon shown in Figure 5, where the excited-state wavepackets cross almost immediately to the potential energy surface of a CT intermediate. We suggested recently^21^ that the damping of excited state wavepackets during hot-carrier cooling arises from *quantum quenching*,^62^ which is effectively a rapid change in the forces acting on the system and on the vibrational displacements that accompany the change in electronic structure due to passage through a CI.^63^ The proposal that the passage of wavepackets from the optically prepared X3 state to the CT state involves a CI between the two states accounts for the finding that the damping of the MV mode accompanies the electronic relaxation time scale. The observation that the initial “quantum beat” is somewhat less intense in the MV^2+^-treated QDs is consistent with a more rapid damping of wavepacket motions during passage through a CI or CIs on the way down from the Franck–Condon excited-state structure. The coherences evidently persist long enough upon crossing to the CT state to contribute to the observed modulations at low detection energies in the 2DES spectra.

**Figure 5:**
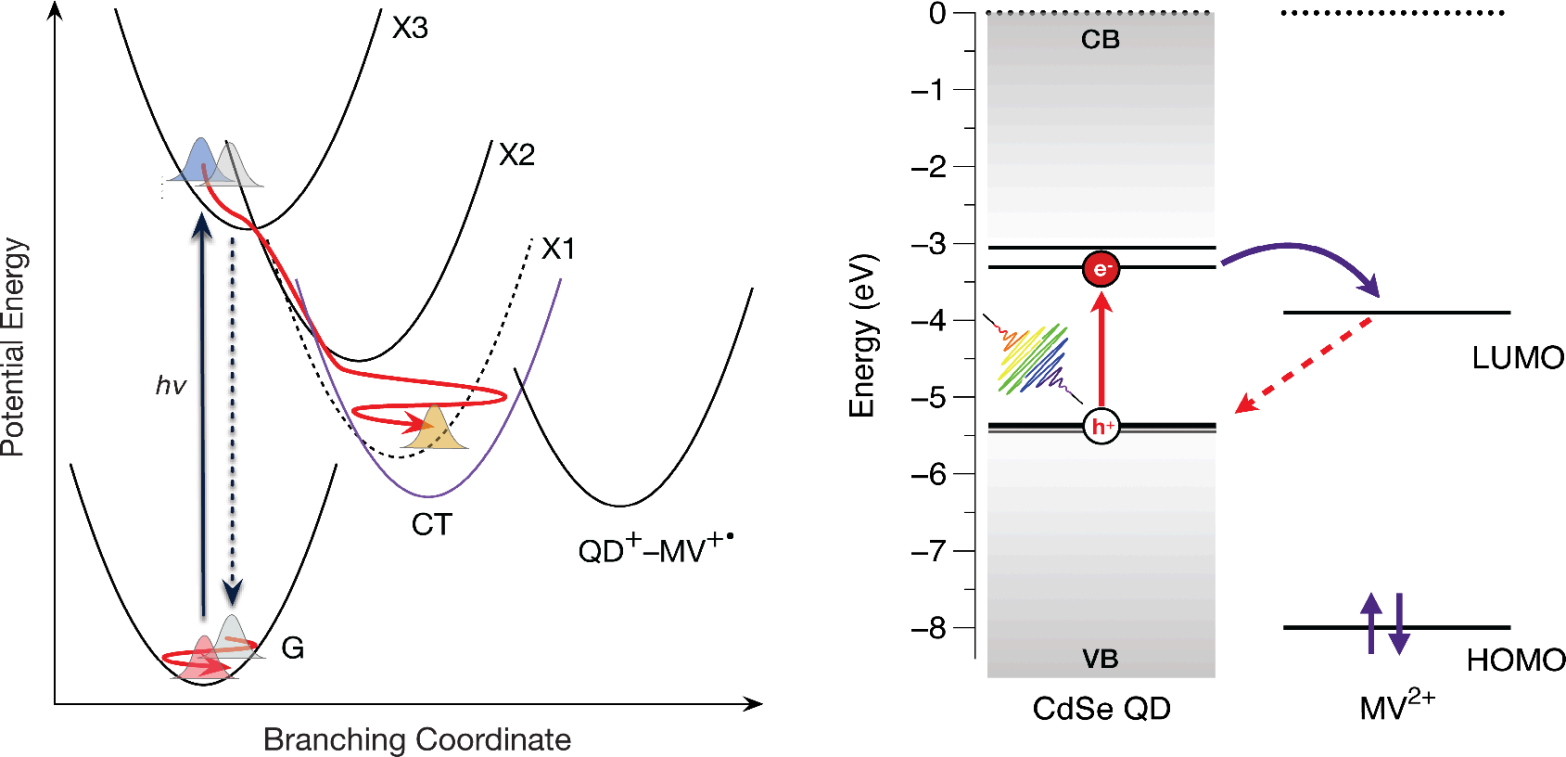
*Left:* Coherent nonadiabatic mechanism for photoinduced CT following optical preparation of the X3 exciton state in the methyl viologen dication (MV^2+^)-treated CdSe QDs. Upon a vibrationally impulsive absorption transition from the equilibrium ground state (G) to reach the Franck–Condon excited state structure, wavepackets are instantaneously displaced with respect to the resonance Raman-active vibrational coordinates. The branching coordinates then promote the passage of wavepackets through one or more conical intersections (CIs) to reach a charge-transfer state (CT) rather than the band-edge X1 state (dashed). The CT state serves as an intermediate, with dephasing and IVR-mediated cooling prior to a crossing to the QD^+^–MV^+•^ product surface. *Right:* Energetics of photoinduced CT from CdSe QDs to MV^2+^, based on a figure for CdS QDs.^34^

We can tell that the CT state here has a mixed QD–MV character, however, because it is borrowing enough oscillator strength from the QD to make it possible to observe the vibrations of the MV^2+^ acceptor. The contribution of the core of the QD is made evident by detection following the initial beat in the (X3,PL) transient of relatively strong recurrent modulations at 210 cm^–1^ with a damping time of 400 fs. This component is readily apparent in the (X3,PL) residual transient shown in Figure 4a. The observed frequency clearly indicates that this arises from the LO phonon, but the shorter damping time observed in the MV^2+^-treated sample shows that these are principally excited-state wavepacket motions, retaining coherence after the passage of wavepackets to the CT state. Keeping in mind that the LPSVD spectrum in Figure 3 reports the power of the modulation components, so the modulation amplitude is proportional to the area under the spectral peak, it is obvious from an inspection of the residual transient that the modulations due to the LO phonon are stronger as well as being more rapidly damped overall in the MV^2+^-treated sample compared to that of the oleate-capped control. These observations suggest that the lattice vibrations of the core are strongly displaced by a partial redistribution of charge from the core of the QD to the MV^2+^ acceptor in the CT state. The damping time of 400 fs is somewhat longer than that assigned above to IVR processes in the global population model, but it is a third of that for the ground state, stimulated Raman coherences in the LO phonon in the control, oleate-capped QDs. This analysis suggests that the local LO phonon mode in the MV^2+^-treated QDs survives the crossing to the CT state as a spectator rather than as a promoting mode for the photoinduced CT mechanism.

## Conclusions

This work builds on the observations made in our recent comparison of the dynamics in CdSe QDs with HDA and oleate surface capping ligands, where we found that the conversion of the excitation energy captured by absorption transitions to the X3 state to ligand vibrations can be detected on a short time scale compared to the subsequent IVR time scale using the information from the damping of vibronic coherences.^21^ The significance of that work includes the prospect that the coherences of electron acceptors adsorbed or bound to the surface of QDs can be exploited to improve the energy efficiency of QD-based devices. The present work is the first study to our knowledge of photoinduced CT in QDs with impulsive excitation, allowing the characterization of the vibronic coherences that are launched upon absorption transitions. The results show that the transition state for the photoinduced CT to MV^2+^ is crossed in <50 fs owing to passage of wavepackets from the optically prepared X3 state to a CT intermediate with retention of vibronic coherence via a coherent nonadiabatic mechanism. These findings emphasize again that we can sense the abrupt change in electronic structure that accompanies the passage through the CIs by characterizing the modulation frequencies and damping times of the excited-state coherences, and here the earlier recurrence of the initial “quantum beat” and of the subsequent modulations at the LO phonon frequency provides clear evidence for a faster electronic process in the MV^2+^-treated QDs that outcompetes the transfer of excitation energy to the oleate ligand vibrations. The coherences evidently provide better information on the reaction mechanism than the global models for the populations, which are capable of providing only an average time scale for the ultrafast processes leading to the product state.

The 415 cm^–1^ vibrational mode observed in the rapidly damped vibronic coherences is a good candidate for a branching mode associated with a CI between the X3 and CT states because of its short damping time and because its out-of-plane character would modulate the transfer of electron density to the MV^2+^ acceptor. However, the presence of resolved cross peaks at very short delays *T* below the diagonal of the 2DES spectrum suggests the possibility that the X2 and X1 states of the QD have obtained some partial CT character as well, which would suggest that the excited-state trajectory from X3 to the CT state might involve passage through more than one CI, perhaps from X3 to X2 or X1 and then to the CT state. A more detailed analysis of the phasing of the vibronic coherences and of the effect of different modes of binding of MV, such as for the phosphonated MV,^64^ might be considered in a future study. The LO phonon is resonance Raman active;^65^ displacement from the Franck–Condon structure in the X3 state initiates the trajectory leading to the CIs with respect to the branching modes.

Importantly, the driving force for the full transfer of an electron to the MV acceptor is actually determined in this picture by the energy of the CT state, which effectively serves as the transition state for the full transfer of an electron. The CT state would serve as a bridge between the locally excited QD and the LUMO energy levels of the MV^2+^ acceptor, using the conventional picture shown in the right-hand panel of Figure 5, which is derived from studies of photoinduced CT in CdS QDs.^34^ Although the electron-transfer time scale is at least an order of magnitude longer than the decoherence time of the CT state, the yield and persistence of the MV^+•^ product is determined by the energy and the couplings of the CT state from the optically prepared exciton state to the MV^+•^ product state. The properties of the CT state are determined by the nature of the electronic interaction of MV^2+^ with the surface Se^2–^ ions assumed upon absorption to the surface of the QD.^66^ This idea is crucial to the eventual optimization of the use of QDs in photocatalysis and in energy conversion applications though the use of the chemistry of surface-bound organic reactants.^3^

## Supplementary Material



## Data Availability

The data sets and the code used to analyze the results reported in this paper will be provided in an archive at the following DOI: 10.5281/zenodo.18404440.

## Notes

Residual phase spectra of the pump and probe excitation pulses; MIIPS SHG–FROG spectrogram and interferometric autocorrelation signals; tables of LPSVD model parameters; and oscillation maps for the oleate-capped and MV^2+^-treated CdSe QDs (PDF)

## #

Applied Materials, Inc., 3050 Bowers Avenue, Santa Clara, California 95054, United States
