# Supplementary material for: Photoinduced Charge Transfer and Vibronic Coherence in CdSe Quantum Dots with Methyl Viologen Acceptors
Source: J Phys Chem C Nanomater Interfaces. 2026 Apr 22;130(18):6580–8. doi: 10.1021/acs.jpcc.6c02147 (PMC13158992; doi:10.1021/acs.jpcc.6c02147)
Supplement: Supplementary file 1 [file jp6c02147_si_001.pdf]

Supplemental Information

# Photoinduced Charge Transfer and Vibronic Coherence in CdSe Quantum Dots with Methyl Viologen Acceptors

Nila Mohan T. M.,[1,a] Shanu A. Shameem,[1] Chase H. Leslie,[1] Caitlin V. Hetherington,[2] Benjamin G. Levine,[2] and Warren F. Beck[1,*]

[1]Department of Chemistry, Michigan State University,
578 S. Shaw Lane, East Lansing, Michigan 48824 U.S.A.

[2]Institute for Advanced Computational Science and Department of Chemistry,
Stony Brook University, Stony Brook, New York 11794 U.S.A.

[a]Current address: Applied Materials, Inc., 3050 Bowers Avenue,
Santa Clara, CA 95054, U.S.A.

*Corresponding author. Email: beckw@msu.edu

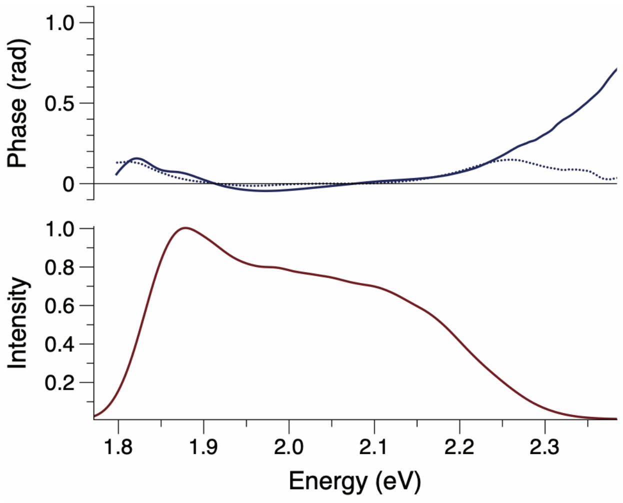


**Figure S1.** Residual phase and intensity spectra of the femtosecond laser pulses used in the broadband multidimensional electronic spectroscopy experiments. *Top panel:* Residual phase spectra after compression of the laser pulses pump beam (blue solid), and for the probe beam (blue dashed), as determined by MIIPS scans.[1] *Bottom panel:* Intensity spectrum of the laser pulses.

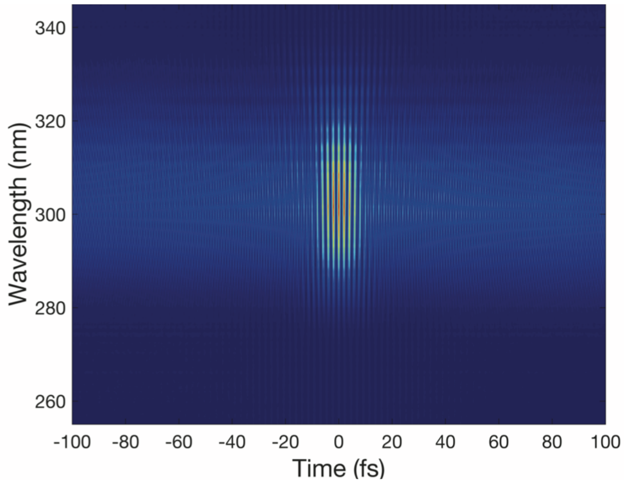


**Figure S2.** SHG-FROG[2] spectrogram for the pump pulses, as measured with scanned pulse pairs prepared by the pulse shaper.

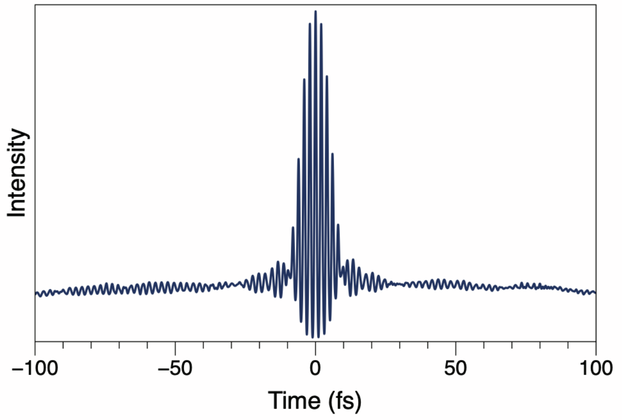


**Figure S3.** Interferometric autocorrelation of the pump pulses, determined as the integral of the SHG-FROG spectrogram, Figure S2.

**Table S1.** LPSVD fit parameters[a] for the fit to the residual transient shown in Figure 4, for the (X3,PL) coordinate in 2DES spectrum from the oleate-capped CdSe QDs.

| $\omega$ ($cm^{-1}$) | $A$ | $\phi$ (deg) | $\gamma$ (fs) |
|---|---|---|---|
| 42.3 | 0.0158236 | 54.9 | 810 |
| 126.3 | 0.171867 | −57.4 | 58.3 |
| 201.5 | 0.0132064 | −5.2 | 1230 |
| 376.6 | 0.455532 | 69.5 | 32.2 |

$a$. Damped cosinusoids, $A\cos(\omega T - \phi)\exp(-T/\gamma)$

**Table S2.** LPSVD fit parameters[a] for the fit to the residual transient shown in Figure 4, for the (X3,PL) coordinate in the 2DES spectrum from the $MV^{2+}$-treated CdSe QDs.

| $\omega$ ($cm^{-1}$) | $A$ | $\phi$ (deg) | $\gamma$ (fs) |
|---|---|---|---|
| 56.8 | 0.00142 | 80.8 | 467.6 |
| 150.2 | 0.0769 | −88.1 | 90.9 |
| 210.3 | 0.0334 | −35.3 | 411.9 |
| 414.3 | 0.347 | 53.4 | 27.2 |

$a$. Damped cosinusoids, $A\cos(\omega T - \phi)\exp(-T/\gamma)$

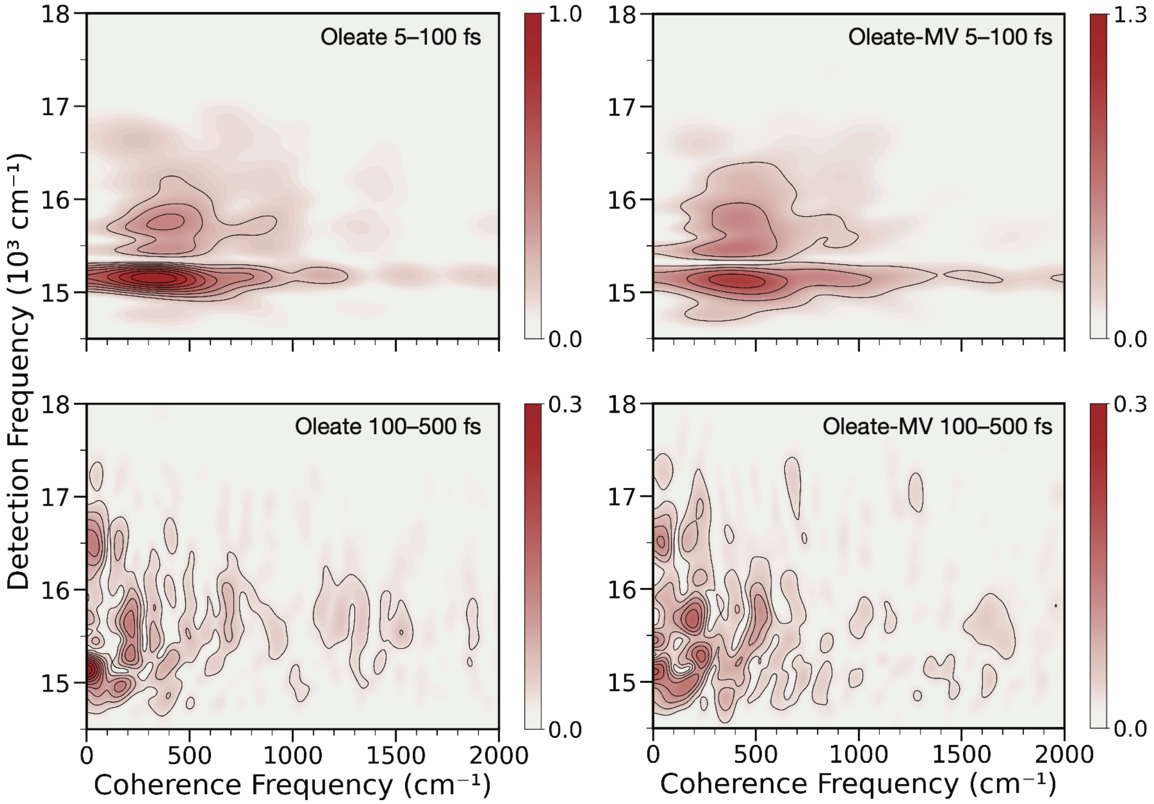


**Figure S4.** Oscillation maps for coherences in the 2DES spectrum of the control oleate-capped and methyl viologen treated CdSe QDs at the X3 (2.2 eV) slice of the excitation axis of the 2DES spectrum. The Fourier transform power spectrum of the 5–100-fs and 100–500-fs windows of the probe delay *T* axis are plotted as a function of the detection energy.

## References


(1) Lozovoy, V. V.; Pastirk, I.; Dantus, M. Multiphoton Intrapulse Interference. IV. Ultrashort Laser Pulse Spectral Phase Characterization and Compensation. *Opt. Lett.* **2004**, *29*, 775–777. DOI: 10.1364/ol.29.000775.

(2) DeLong, K. W.; Trebino, R.; Hunter, J.; White, W. E. Frequency-Resolved Optical Gating with the Use of Second-Harmonic Generation. *J. Opt. Soc. Am. B* **1994**, *11*, 2206–2215. DOI: 10.1364/JOSAB.11.002206.
